# Association between coenzyme Q 10-related genetic polymorphisms and statin-associated myotoxicity in Korean stroke patients

**DOI:** 10.3389/fphar.2024.1358567

**Published:** 2024-05-07

**Authors:** Yoon-A Park, Yoonkyung Chang, Da Hoon Lee, Jung Sun Kim, Minju Park, Seo-A Choi, Tae-Jin Song, Hye Sun Gwak

**Affiliations:** ^1^ College of Pharmacy and Graduate School of Pharmaceutical Sciences, Ewha Womans University, Seoul, Republic of Korea; ^2^ Department of Neurology, Mokdong Hospital, College of Medicine, Ewha Womans University, Seoul, Republic of Korea; ^3^ Department of Neurology, Seoul Hospital, College of Medicine, Ewha Womans University, Seoul, Republic of Korea

**Keywords:** statin, myotoxicity, coenzyme Q 10, polymorphisms, pharmacogenomics

## Abstract

**Introduction:**

The purpose of this study is to identify the relationship between coenzyme Q 10 (CoQ10)-related gene polymorphisms and statin-related myotoxicity (SRM).

**Methods:**

We retrospectively analyzed prospectively collected samples from February to May 2021. To investigate the association between CoQ10-related genetic factors and SRM, we selected 37 single nucleotide polymorphisms from five genes (*COQ2, COQ3, COQ5, COQ6*, and *COQ7*). The odds ratio (OR) and adjusted OR with 95% confidence intervals (CI) were calculated for univariate and multivariable logistic regression analyses, respectively.

**Results:**

A total of 688 stroke patients were included in the analysis, including 56 SRM cases. In the multivariable analysis, two models were constructed using demographic factors only in model I, and demographic and genetic factors in model II. Compared to other statins, atorvastatin decreased the SRM risk whereas ezetimibe use increased the SRM risk in model I and model II. Patients with *COQ2* rs4693075 G allele, *COQ3* rs11548336 TT genotype, and *COQ5* rs10849757 A allele had a 2.9-fold (95% CI: 1.6–5.3), 1.9-fold (95% CI: 1.1–3.5), and 3.3-fold (95% CI: 1.5–8.3) higher risk of SRM, respectively.

**Conclusion:**

This study could be utilized to develop a personalized medicine strategy in patients treated with statins.

## Introduction

Hydroxymethylglutaryl-coenzyme A (HMG-CoA) reductase inhibitors, known as statins, have been used as first-choice drugs for the primary and secondary prevention of cardiovascular diseases (Adhyaru and Jacobson, 2018). Numerous studies and guidelines have emphasized the importance of statin use. A recent meta-analysis reported that the use of statins in patients with increased cardiovascular disease risk reduced their risk for all-cause mortality by 0.9-fold and cardiovascular events by 0.7-fold (Chou et al., 2022). The 2019 American College of Cardiology/American Heart Association guideline recommended that patients with high atherosclerotic cardiovascular disease (ASCVD) risk should be treated with statins to reduce the risk. High-intensity statin therapy should be initiated to prevent ASCVD when adult patients’ low-density lipoprotein (LDL) cholesterol levels are not less than 190 mg/dL (Arnett et al., 2019).

Statin therapy is effective and tolerable; however, problems with statin toxicity remain. Of all adverse events related to statins, statin-related myotoxicity (SRM) is the most common and occurs in approximately 10%–25% of cases (Vinci et al., 2021). SRM phenotypes vary, ranging from mild creatine kinase (CK) elevation to severe rhabdomyolysis. SRM also includes myalgia, muscle cramps, myopathy, and immune-mediated statin myopathy (Turner and Pirmohamed, 2019).

There are several hypotheses of musculoskeletal adverse events. Several studies suggested that mitochondrial dysfunction and coenzyme Q 10 (CoQ10) depletion caused by statins may be associated with SRM (De Pinieux et al., 1996). CoQ10 is produced by the mevalonate pathway and functions as an electron carrier in the mitochondrial electron transfer system, which protects against reactive oxygen species (Dohlmann et al., 2022). Because statins inhibit HMG-CoA reductase, which participates in the mevalonate pathway, CoQ10 levels might become decreased in the muscles leading to adverse events like muscle cramps, myalgia, and others (Kennedy et al., 2020).

CoQ10 is synthesized step by step via the mevalonate pathway and numerous genes are required for its biosynthesis (Doimo et al., 2014). Several studies analyzed the association between CoQ10-related genetic variants and various diseases induced by decreased CoQ10 levels (Mantle et al., 2023). Among them, *COQ2* rs4693075 was mainly studied, but the previous studies were limited and controversial (Oh et al., 2007; Puccetti et al., 2010; Carr et al., 2013; Hubacek et al., 2017; Bakar et al., 2018; Ramakumari et al., 2018; Chowdhury et al., 2019). Moreover, no study has investigated the effect of other CoQ10-related genetic variants on SRM. Therefore, this study aimed to identify the association between SRM and genetic factors related to CoQ10 biosynthesis including *COQ2, COQ3, COQ5, COQ6*, and *COQ7* in Korean stroke patients receiving statins.

## Materials and methods

### Study patients

We performed a retrospective analysis of prospectively collected samples from February to May 2021 at Ewha Womans University Seoul Hospital and Ewha Womans University Mokdong Hospital. This study was approved by the Institutional Review Board (IRB) of each hospital in agreement with the 1975 Declaration of Helsinki and its later amendments (IRB numbers: 2020-11-014 and 2021-02-026, respectively) Written informed consent was obtained from all patients before enrollment.

The inclusion criteria of patients of this study were those aged not less than 20 years old who had been treated with statins (atorvastatin, rosuvastatin, pitavastatin, pravastatin, or simvastatin) for the secondary prevention for ASCVD after stroke. Patients administered statins for at least 4 weeks were included in the control group. Cases of myotoxicity were defined by the following criteria: (Adhyaru and Jacobson, 2018): intolerable myalgia with CK < 4 × upper limit of normal (ULN) (SRM 2); (Chou et al., 2022); myopathy with a CK level between 4 × ULN and 10 × ULN (SRM 3); or (Arnett et al., 2019) severe myopathy with a CK level between 10 × ULN and 50 × ULN (SRM 4) (Alfirevic et al., 2014). The ULN for CK levels was 120 U/L (Neal et al., 2009). Patients were excluded if they met the following criteria: (Adhyaru and Jacobson, 2018): they did not have muscle symptoms with CK elevation less than 4 × ULN (SRM 0), (Chou et al., 2022), had tolerable myalgia (SRM 1), (Arnett et al., 2019), had CK not less than 4 × ULN at baseline, (Vinci et al., 2021), had elevated CK or muscle pain within 7 days of other illnesses (surgery, cancer, or heart disease like myocardial infarction), or (Turner and Pirmohamed, 2019) their DNA samples were insufficient for analysis.

We collected data from electronic medical records, including patients’ sex, age, weight, body mass index, total cholesterol, triglyceride, low-density lipoprotein, high-density lipoprotein, CK, smoking, alcohol use, comorbidity, concomitant drugs, and class of statin.

### Genotyping methods

Five candidate genes (*COQ2, COQ3, COQ5, COQ6*, and *COQ7*) were selected to investigate the relationship between CoQ10-related genetic associations and SRM. A total of 37 single nucleotide polymorphisms (SNPs) were chosen based on previous findings (Acosta et al., 2016; Stefely and Pagliarini, 2017; Cunningham et al., 2022), and minor allele frequencies and linkage disequilibrium were determined in Asian populations (Barrett et al., 2005; Ward and Kellis, 2016). We excluded SNPs having the relationship of LD in Asian populations (*r* ^2^ ≥ 0.8) based on HaploReg.

DNA was extracted from patient saliva or blood. We extracted the DNA from blood samples with the QIAamp DNA Blood Mini Kit (QIAGEN, Hilden, Germany) or from saliva with OraGene-600 kits (DNA Genotek, OTT, Canada) and PrepIT reagents (DNA Genotek, OTT, Canada). All SNPs were identified as dbSNP rsID and analyzed by TaqMan genotyping assay. The TaqMan allele discrimination technique was used to perform RT-PCR on ABI 7300 instrument (Applied Biosystems, Carlsbad, CA, United States of America). The PCR was performed in a 25 μL optical 8-cap strip containing 0.2 ng/ μ L of DNA samples and 13.75 μL of PCR mix. The PCR reagent mixture included 12.5 μL of the TaqMan Genotyping Master Mix and 1.25 μL of the 20X TaqMan SNP Genotyping Assay Mix (Applied Biosystems in Foster City, California, United States of America). The catalog number of used assay was 4351379. Ten minutes after denaturing at 95°C, the PCR was run for 15 s at 92 °C for 40 cycles and 60 s at 60°C for 40 cycles.

### Statistical analysis

The chi-squared and Fisher’s exact tests were used to compare the categorical variables between patients who underwent SRM and those who did not. The unpaired *t*-test was used to compare continuous variables. Multivariable logistic regression analysis with backward elimination was used to identify the independent risk factors for SRM using variables with *p* < 0.05 in the univariate analysis, including sex and age. Two models were constructed using demographic factors only (model I), and demographic and genetic factors (model II). The unadjusted and adjusted odds ratios (ORs) with 95% confidence intervals (CI) were calculated from univariate and multivariable analyses, respectively. Haplotype analysis was carried out on gene SNPs exhibiting significance in the multivariable analysis using Haploview software (version 4.2; Broad Institute of Massachusetts Institute of Technology and Harvard University, Cambridge, MA, United States of America). The Hosmer-Lemeshow goodness-of-fit test was performed for the fit of the prediction model. The discrimination of the model was evaluated further by calculating the area under the receiver operating characteristic curve (AUROC). Sensitivity analysis was conducted to evaluate outcomes by adding patients with SRM 0 or SRM 1 into the case group. All statistical analyses were performed using R software (version 4.2.2; R Foundation for Statistical Computing, Vienna, Austria). *p* < 0.05 was considered statistically significant.

## Results

**FIGURE 1:**
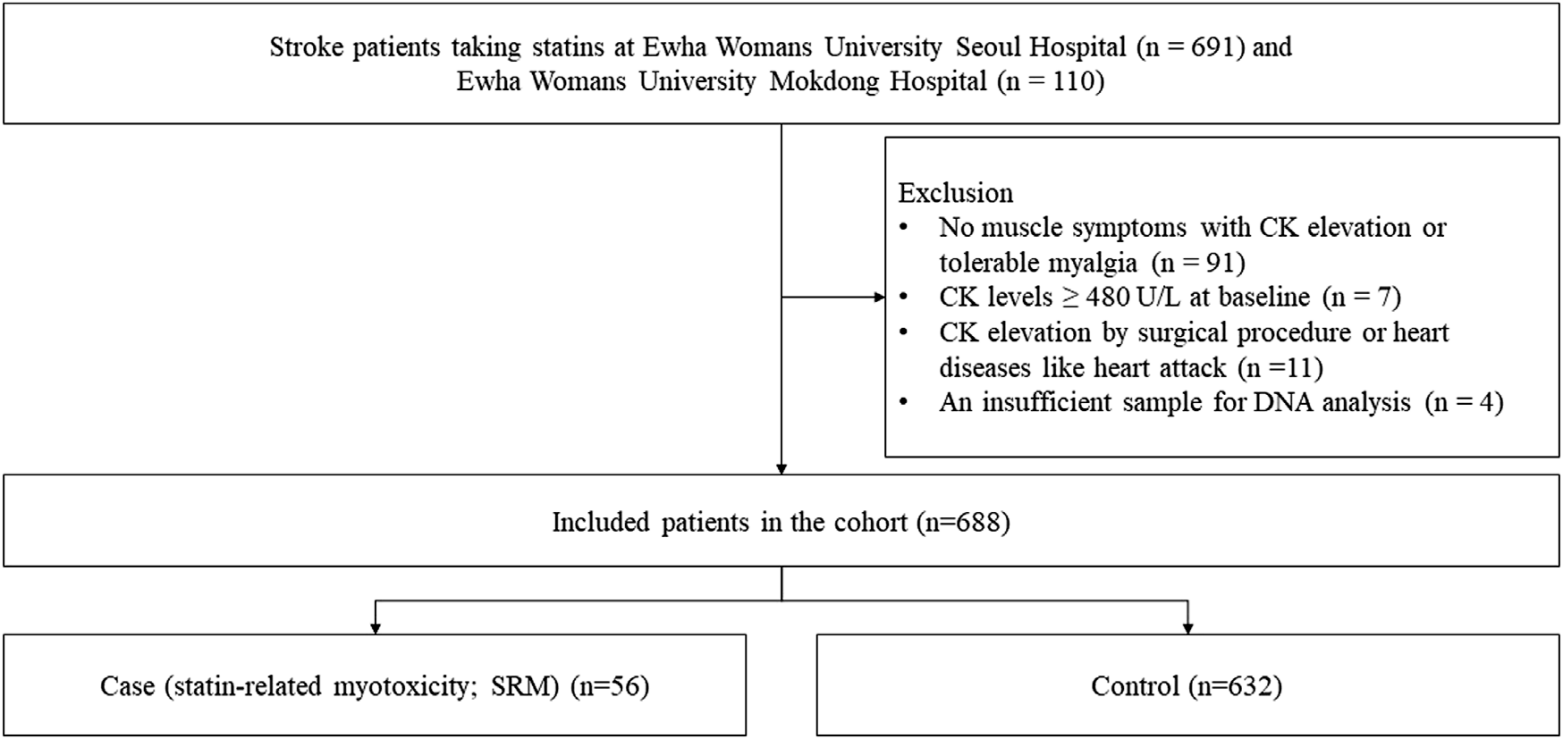
Flow chart of patient selection.

A total of 801 stroke patients were enrolled in the study (Figure 1). We excluded 91 patients who did not have muscle symptoms with CK elevation or had tolerable myalgia without CK elevation. Seven patients were excluded because their baseline CK levels were not less than 480 U/L. Eleven patients were excluded because their CK levels were elevated by other medical issues including myocardial infarction and surgical procedures. We also excluded four patients as their DNA samples were insufficient for analysis. As a result, 688 patients were included in the analysis. Of the included patients, 56 experienced statin-associated muscle symptoms. Twelve patients underwent myopathy (SRM 3) after statin treatment, and two patients experienced severe myopathy (SRM 4). Forty-two patients had intolerable myalgia (SRM 2). The remaining 632 patients who never experienced SRM were classified as the control group.

In the case group comprising 56 patients experiencing SRM, three patients (5.4%) discontinued statin therapy, while 27 patients (48.2%) transitioned to alternative statins. Among the nine patients initially prescribed atorvastatin, three switched to rosuvastatin, five to pitavastatin, and one to pravastatin. Within the cohort of 15 patients receiving rosuvastatin, nine shifted to atorvastatin, two to pitavastatin, and one to pravastatin. Of the three patients using pitavastatin, one transitioned to atorvastatin, one to rosuvastatin, and the remaining patient to pravastatin. All patients exhibited good tolerance to the substituted statins. Moreover, seven patients (12.5%) in total underwent dose reduction of statins, with six patients demonstrating good tolerance to the adjusted doses. In the control group, none experienced SRM, and all participants exhibited good tolerance to statin therapy.

The demographic and clinical characteristics of the included patients are presented in Table 1. The mean age of the study population was 63.1 years, and male patients comprised 69.6% of the cohort. The most administered statin was atorvastatin (53.3%), followed by rosuvastatin (39.0%). The duration of therapy in the SRM group was shorter than in the control group (520.1 days vs. 828.5 days, *p* = 0.004). The most co-medicated drugs were antiplatelet drugs (84.3%) followed by angiotensin-converting enzyme inhibitors or angiotensin II receptor blockers (52.6%). Statin types, ezetimibe, and diuretics were significant factors for SRM. The CK values according to genotypes are presented in the Supplementary Table S1.

**TABLE 1:** Baseline characteristics of patients included in this study.

Characteristics	Statin-related myotoxicity (n = 56)	Control (n = 632)	Total (n = 688)	*p*
Sex				0.451
Male	36 (64.3)	443 (70.1)	479 (69.6)	
Female	20 (35.7)	189 (29.9)	209 (30.4)	
Age (years)	62.7 ± 10.9	63.2 ± 11.0	63.1 ± 11.0	0.776
Weight (kg)	65.4 ± 9.2	66.4 ± 11.4	66.3 ± 11.3	0.455
Body mass index (kg/m2)	24.5 ± 2.7	24.4 ± 3.2	24.4 ± 3.2	0.827
Comorbidities				
Hypertension	37 (66.1)	452 (71.5)	489 (71.1)	0.479
Diabetes mellitus	19 (33.9)	206 (32.6)	225 (32.7)	0.956
Dyslipidemia	25 (44.6)	322 (50.9)	347 (50.4)	0.444
Congestive heart failure	0 (0.0)	15 (2.4)	15 (2.2)	0.491
Atrial fibrillation	9 (16.1)	87 (13.8)	96 (14.0)	0.783
Myocardial infarction	1 (1.8)	16 (2.5)	17 (2.5)	1
Cancer	4 (7.1)	35 (5.5)	39 (5.7)	0.844
Chronic liver diseases	2 (3.6)	18 (2.8)	20 (2.9)	1
Chronic kidney diseases	1 (1.8)	19 (3.0)	20 (2.9)	0.915
Hypothyroidism	1 (1.8)	19 (3.0)	20 (2.9)	0.915
Smoking	16 (29.1)	245 (39.0)	261 (38.2)	0.191
Alcohol	20 (36.4)	271 (43.2)	291 (42.6)	0.404
Lipid profile				
Total cholesterol (mg/dL)	179.1 ± 52.4	178.9 ± 43.7	178.9 ± 44.4	0.975
Triglyceride (mg/dL)	129.5 ± 63.7	128.0 ± 76.3	128.1 ± 75.3	0.896
LDL (mg/dL)	110.7 ± 43.3	111.0 ± 35.9	111.0 ± 36.4	0.948
HDL (mg/dL)	49.3 ± 11.3	46.2 ± 10.9	46.4 ± 10.9	0.08
eGFR-CKD-EPI (mL/min/1.73 $m^{2}$ )	82.8 ± 18.8	83.9 ± 17.7	83.8 ± 17.8	0.686
AST	28.0 ± 10.5	27.5 ± 22.2	27.6 ± 21.5	0.777
ALT	27.2 ± 20.2	24.3 ± 26.6	24.5 ± 26.1	0.34
Creatine kinase (U/L)	154.3 ± 34.6	106.9 ± 57.1	110.2 ± 66.6	0.035
Type of statin				0.009
Atorvastatin	18 (32.1)	349 (55.2)	367 (53.3)	
Rosuvastatin	30 (53.6)	238 (37.7)	268 (39.0)	
Other statins^a^	8 (14.3)	45 (7.1)	53 (7.7)	
Comedication				
Ezetimibe	15 (26.8)	81 (12.8)	96 (14.0)	0.007
Fibrate	0 (0.0)	5 (0.8)	5 (0.7)	1
ACEI/ARB	31 (55.4)	331 (52.4)	362 (52.6)	0.773
Beta blockers	7 (12.5)	90 (14.2)	97 (14.1)	1
Calcium channel blockers	15 (26.8)	242 (38.3)	257 (37.4)	0.118
Diuretics	12 (21.4)	64 (10.1)	76 (11.0)	0.018
Antiplatelet drugs^b^	42 (75.0)	538 (85.1)	580 (84.3)	0.071
Anticoagulants^c^	8 (14.3)	101 (16.0)	109 (15.8)	0.887
Duration of therapy (days)	520.1 ± 654.3	828.5 ± 773.4	803.8 ± 768.7	0.004

^a^ pravastatin (1.0%), pitavastatin (5.2%), or simvastatin (1.5%).

^b^ aspirin, clopidogrel, cilostazole, sarpogrelate, and triflusal.

^c^ apixaban, dabigatran, edoxaban, rivaroxaban, and warfarin.; ACEI: angiotensin converting enzyme inhibitor; ALT: alanine transaminase; ARB: angiotensin II, receptor blocker; AST: aspartate aminotransferase; eGFR-CKD-EPI: estimated glomerular filtration rate-chronic kidney disease-epidemiology collaboration; HDL: high-density lipoprotein; LDL: low-density lipoprotein.

Table 2 shows the association between CoQ10-related gene SNPs and SRM. Wild-type allele (G) carriers of *COQ2* rs4693075 had a higher risk of SRM than mutant-type homozygote carriers (CC) (13.2% vs. 6.3%, *p* = 0.006). Regarding *COQ3* genes, rs11548336 was significantly associated with statin-associated muscle symptoms. Mutant-type allele (C) carriers of rs11548336 had a lower risk of SRM than wild-type homozygote (TT) carriers (6.5% vs. 12.0%, *p* = 0.023). Variant allele (A) carriers of *COQ5* rs10849757 were more associated with SRM risk than wild-type homozygote carriers (GG) (9.7% vs. 3.9%, *p* = 0.024).

**TABLE 2:** Effects of *COQ2*, *COQ3*, *COQ5*, and *COQ7* grouped genotypes on statin-related myotoxicity.

dbSNP rsID	Minor allele frequency in Asians	Grouped genotype	Statin-related myotoxicity (n = 56)	Control (n = 632)	*p*
*COQ2*					
rs4693075 (G>C)	0.13	GG, CG	24 (42.9)	158 (25.1)	0.006
		CC	32 (57.1)	472 (74.9)	
rs121918233 (C>T)	<0.01	CC	56 (100.0)	632 (100.0)	
rs145182498 (G>A)	0.02	GG	55 (98.2)	604 (96.0)	0.648
		AA, AG	1 (1.8)	25 (4.0)	
rs745504932 (C>T)	<0.01	CC	56 (100.0)	631 (99.8)	1
		TT, CT	0 (0.0)	1 (0.2)	
rs121918230 (T>C)	<0.01	TT	48 (96.0)	568 (97.6)	0.826
		CC, CT	2 (4.0)	14 (2.4)	
rs121918231 (C>T)	<0.01	CC	56 (100.0)	631 (100.0)	
rs121918232 (T>C)	<0.01	TT	56 (100.0)	624 (99.5)	1
		CC, CT	0 (0.0)	3 (0.5)	
rs34110644 (G>A)	0.31	GG, AG	31 (55.4)	319 (50.6)	0.591
		AA	25 (44.6)	311 (49.4)	
rs6849677 (T>C)	0.01	TT, CT	0 (0.0)	10 (1.6)	0.714
		CC	56 (100.0)	621 (98.4)	
rs761785906 (G>A)	<0.01	GG	56 (100.0)	630 (100.0)	
rs867410805 (C>T)	<0.01	CC	56 (100.0)	631 (100.0)	
*COQ3*					
rs6925344 (T>C)	0.18	TT, CT	21 (37.5)	168 (26.7)	0.113
		CC	35 (62.5)	462 (73.3)	
rs11548336 (T>C)	0.44	TT	25 (44.6)	184 (29.1)	0.023
		CC, CT	31 (55.4)	448 (70.9)	
rs146934336 (G>A)	<0.01	GG	56 (100.0)	631 (100.0)	
rs376598849 (C>T)	<0.01	CC	56 (100.0)	631 (100.0)	
rs200092962 (C>T)	<0.01	CC	56 (100.0)	632 (100.0)	
rs6912105 (A>G)	0.46	AA, AG	42 (75.0)	435 (68.9)	0.428
		GG	14 (25.0)	196 (31.1)	
rs769495529 (C>T)	<0.01	CC	56 (100.0)	631 (100.0)	
rs9389319 (A>C)	0.22	AA	8 (14.3)	40 (6.3)	0.05
		CC, AC	48 (85.7)	590 (93.7)	
rs9483838 (G>A)	0.38	GG	17 (30.4)	226 (35.9)	0.496
		AA, AG	39 (69.6)	404 (64.1)	
*COQ5*					
rs3742049 (C>T)	0.08	CC	46 (82.1)	532 (84.6)	0.773
		TT, CT	10 (17.9)	97 (15.4)	
rs10849757 (G>A)	0.48	GG	7 (12.5)	172 (27.3)	0.024
		AA, AG	49 (87.5)	458 (72.7)	
rs14017 (T>C)	<0.01	TT	56 (100.0)	629 (100.0)	
rs144115488 (C>A)	<0.01	CC	56 (100.0)	628 (99.8)	1
		AA, AC	0 (0.0)	1 (0.2)	
rs4766965 (G>A)	0.04	GG	51 (91.1)	597 (94.5)	0.459
		AA, AG	5 (8.9)	35 (5.5)	
rs1671766 (C>A)	0.28	CC, AC	56 (100.0)	588 (93.0)	0.079
		AA	0 (0.0)	44 (7.0)	
rs758118388 (A>T)	<0.01	AA	56 (100.0)	631 (100.0)	
rs776253786 (C>T)	<0.01	CC	56 (100.0)	632 (100.0)	
*COQ6*					
rs8500 (G>A)	0.23	GG, AG	55 (98.2)	587 (93.2)	0.234
		AA	1 (1.8)	43 (6.8)	
rs2074930 (A>T)	0.34	AA	30 (53.6)	281 (44.7)	0.254
		TT, AT	26 (46.4)	348 (55.3)	
*COQ7*					
rs138730205 (G>C)	0.01	GG	55 (98.2)	614 (97.3)	1
		CC, CG	1 (1.8)	17 (2.7)	
rs4782202 (A>G)	0.02	AA, AG	1 (1.8)	26 (4.1)	0.615
		GG	55 (98.2)	605 (95.9)	
rs11074359 (C>T)	0.17	CC, CT	14 (25.0)	213 (33.8)	0.235
		TT	42 (75.0)	418 (66.2)	
rs74841864 (G>A)	0.06	GG	52 (92.9)	550 (87.0)	0.292
		AA, AG	4 (7.1)	82 (13.0)	
rs77337400 (C>T)	0.02	CC	49 (87.5)	586 (92.9)	0.233
		TT, CT	7 (12.5)	45 (7.1)	
rs72777502 (C>T)	<0.01	CC	56 (100.0)	631 (100.0)	
rs864321686 (T>A)	<0.01	TT	56 (100.0)	631 (100.0)	

Two models were constructed for multivariable logistic regression analysis using factors with *p* < 0.05 in the univariate analysis, along with age and sex (Table 3). Model I was constructed based on clinical factors only, and model II was based on clinical factors and genetic factors. For model 1, patients treated with rosuvastatin and other statins (pravastatin, pitavastatin, or simvastatin) had a 2.2- and 3.1-fold higher risk of myotoxicity than those treated with atorvastatin after covariates were adjusted, respectively. Among concomitant drugs, ezetimibe and diuretics had more than 2-fold higher SRM risk than those who were not. As shown in model II, statin type and ezetimibe remained significant factors even after adjusting for genetic factors. Among the SNPs studied, *COQ2* rs4693075, *COQ3* rs11548336, and *COQ5* rs10849757 were significantly associated with SRM risk. Patients carrying the G allele of rs4693075 experienced a 2.9-fold increase in SRM compared with those carrying the CC genotype (95% CI: 1.6–5.3). Patients carrying the TT genotype of rs11548336 and A allele of rs10849757 had a 1.9-fold (95% CI: 1.1–3.5) and 3.3-fold (95% CI: 1.5–8.3) higher risk of muscle-related toxicity, respectively.

**TABLE 3:** Univariate and multivariable regression analyses to identify predictors for statin-related myopathy.

Variables	Unadjusted OR (95% CIs)	Model I	Model II
		Adjusted OR (95% CIs)	Adjusted OR (95% CIs)
Female	1.30 (0.73–2.31)		
Age ≥65	0.72 (0.41–1.27)		
Statin			
Atorvastatin	1.00	1.00	1.00
Rosuvastatin	2.44 (1.34–4.56)	2.15 (1.16–4.07)*	2.22 (1.18–4.30)*
Other statins	3.45 (1.35–8.16)	3.11 (1.20–7.45)*	3.86 (1.44–9.69) **
Ezetimibe	2.49 (1.32–4.70)	2.05 (1.03–3.90)*	2.60 (1.27–5.17) **
Diuretics	2.42 (1.22–4.82)	2.23 (1.06–4.39)*	1.93 (0.88–3.95)
*COQ2* rs4693075 GG, CG	2.24 (1.28–3.92)		2.92 (1.60–5.31) ***
*COQ3* rs11548336 TT	1.96 (1.13–3.42)		1.93 (1.07–3.46)*
*COQ5* rs10849757 AA, AG	2.63 (1.17–5.92)		3.26 (1.48–8.27) **

*p < 0.05; **p < 0.01; ***p < 0.001.

To identify genetic associations between haplotypes and SRM, further haplotype analyses were conducted on genes exhibiting significance in the multivariable analysis. As shown in Supplementary Figure S1, the following SNPs were in a high relationship of LD: rs4693075 and rs34110644 for *COQ2* gene; rs9483838, rs6925344, and rs11548336 for *COQ3* gene; rs1671766 and rs10849757 for *COQ5* gene. There were significant differences between case and control groups in the following haplotype frequencies, which carry alleles demonstrated in the multivariable analysis: *COQ2* CG, GG haplotype; *COQ3* GTT haplotype; *COQ5* CA, and AG haplotype (Supplementary Table S2). *COQ2* GG (rs4693075 and rs34110644) haplotype, *COQ3* GTT (rs9483838, rs6925344, and rs11548336) haplotype, and *COQ5* CA (rs1671766 and rs10849757) haplotype were highly associated with SRM risk.

**FIGURE 2:**
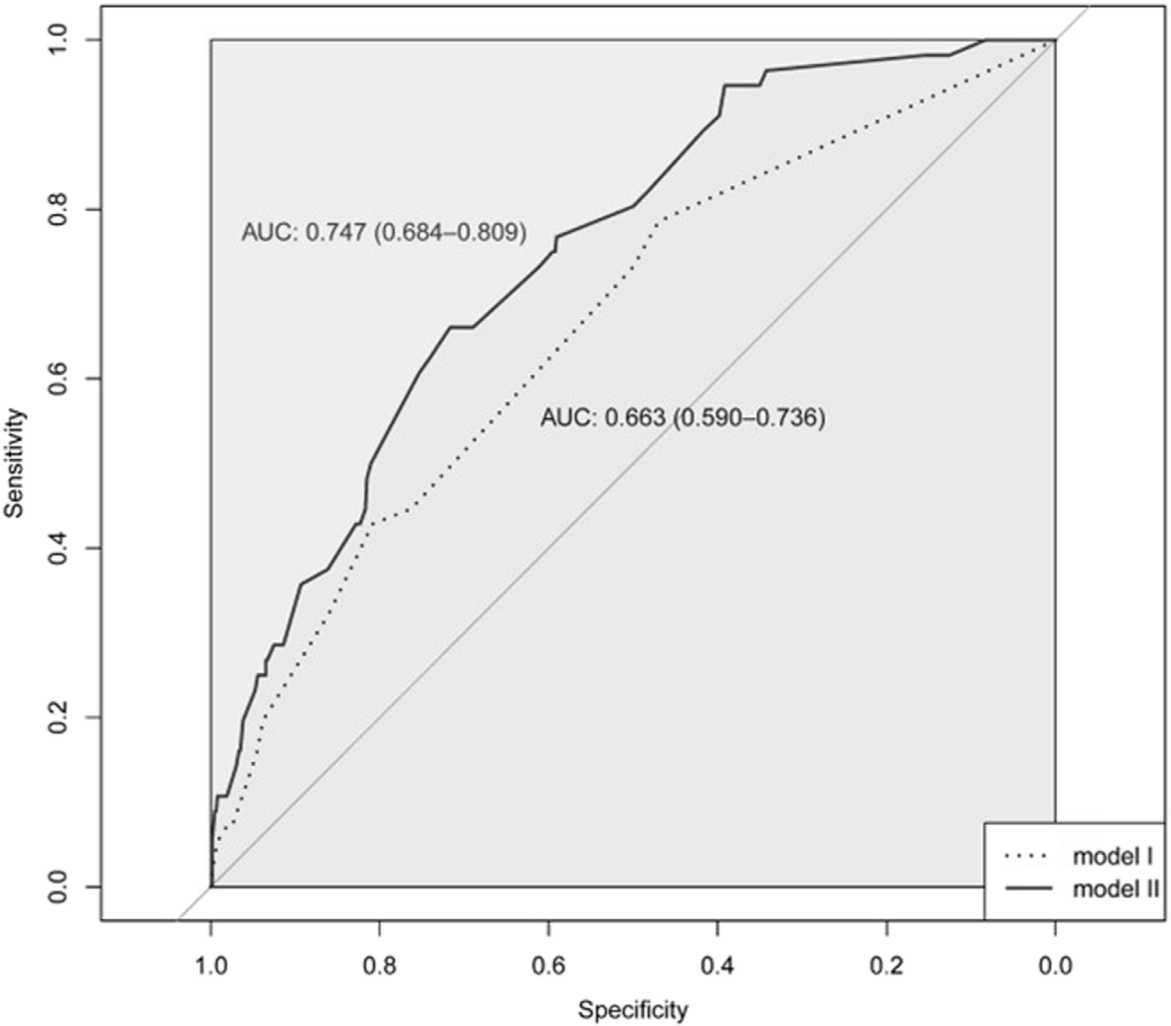
The receiver operating characteristic (ROC) curve for model I and model II.

The Hosmer-Lemeshow test showed that model I and model II were a good fit ( $x^{2}$ = 0.600 and *p* = 0.741; $x^{2}$ = 9.075 and *p* = 0.247, respectively). The AUROC values were 0.663 (95% CI: 0.590–0.736) for model I and 0.747 (95% CI: 0.684–0.809) for model II (Figure 2). We also performed a sensitivity analysis, adding 91 patients (73 with SRM 0 and 18 with SRM 1) into the case group. The final model, in which the same variables as in model II were entered, showed the same trend as the original model: adjusted ORs were 1.76 (95% CI: 1.17–2.67) for rosuvastatin, 2.37 (95% CI: 1.17–4.62) for other statins, 3.04 (95% CI: 1.91–4.82) for ezetimibe, and 1.71 (95% CI: 0.99–2.88) for diuretics. In the case of SNPs, the adjusted ORs were 2.00 (95% CI: 1.32–3.02) for the rs4693075 G allele, 1.79 (95% CI: 1.21–2.65) for the rs11548336 TT genotype, and 2.20 (95% CI: 1.36–3.69) for the rs10849757 A allele.

## Discussion

CoQ 10 is an essential cofactor protecting against oxidative stress in mitochondria and is endogenously synthesized through the mevalonate pathway (Molyneux et al., 2008). It is well known that statins reduce CoQ 10 levels, thereby inducing myotoxicity (Apostolopoulou et al., 2015). Additionally, mutation of *COQ* genes has been correlated with diminished CoQ10 levels. Several case reports have demonstrated that patients with *COQ2, COQ5*, or *COQ7* mutations had decreased CoQ10 levels alongside muscle-related disorders (Jakobs et al., 2013; Malicdan et al., 2018; Wang et al., 2022). This study demonstrated that the CoQ10-related gene polymorphisms, *COQ2* rs4693075, *COQ3* rs11548336, and *COQ5* rs10849757, were significantly related to statin-induced musculoskeletal adverse events. Patients administered rosuvastatin had a higher incidence of SRM than those administered atorvastatin. The concurrent administration of ezetimibe also increased SRM risk compared to no use.

The *COQ2, COQ3, COQ5, COQ6,* and *COQ7* genes encode enzyme synthesizing CoQ10 in eukaryotic mitochondria, which are responsible for rate-limiting steps in the mevalonate pathway (Acosta et al., 2016). COQ2 enzymes condense isoprene to a benzoquinone followed by methylation by COQ3, decarboxylation by COQ5, hydroxylation by COQ6, and deamination by COQ7. Genes involved in the mevalonate pathway have been studied for CoQ10 deficiency, including SRM (Potgieter et al., 2013). For the *COQ2* gene, Oh *et al.* reported that patients with the wild-type homozygote had a 2.3-fold higher risk of SRM than those with the variant-type allele (Oh et al., 2007). Our research revealed a similar result, indicating that the incidence of SRM was higher in patients with the wild-type allele than in those with the variant-type homozygote. However, several studies have reported conflicting results, in which there was no significant association between rs4693075 and statin intolerance, or that the variant allele had a higher risk (Puccetti et al., 2010; Carr et al., 2013; Hubacek et al., 2017; Bakar et al., 2018; Ramakumari et al., 2018; Chowdhury et al., 2019). Therefore, further studies are required to examine ethnic differences in the incidence of SRM.

The present study found that *COQ3* rs11548336 and *COQ5* rs10849757 were associated with SRM. Patients with wild-type alleles of *COQ3* rs11548336 had a higher risk of SRM than mutant allele carriers, which might be due to lower gene expression by the wild-type allele in skeletal muscles, according to the GTEx portal (GTEx ConsortiumLaboratory Data Analysis &Coordinating Center LDACC—Analysis Working GroupStatistical Methods groups—Analysis Working GroupEnhancing GTEx eGTEx groupsNIH Common FundNIH/NCI et al., 2017). rs10849757 is an intron variant of the *COQ5* gene (Ward and Kellis, 2016), and the variant allele increased the SRM risk in this study as opposed to the results that variant alleles showed the highest gene expression in skeletal muscle tissues according to the GTEx portal. CoQ10 deficiency caused by a mutation in the *COQ5* gene was investigated recently, and the lack of COQ5 biosynthesis led to decreased CoQ10 concentrations in skeletal muscles (Malicdan et al., 2018). No studies have investigated the association between rs10849757 of *COQ5* and SRM but considering the possibilities of changing the extent of gene expression by intron variants (Barrett et al., 2012), further studies should be investigated.

As we selected stroke patients for the study, most patients in the cohort were treated with atorvastatin or rosuvastatin. A high dose of atorvastatin and rosuvastatin is a strongly effective therapy for the secondary prevention of ASCVD among ischemic stroke patients (Kleindorfer et al., 2021). A recent randomized controlled trial proved that highly intensive statin treatment was effective at lowering lipid levels among patients after stroke, and this strong evidence was the basis for the statin treatment guidelines (Amarenco et al., 2020). However, the most prevalent adverse event, SRM, is a major issue for patients treated with statins (Backes et al., 2017). The incidence of SRM varies depending on statin classes. Sakaeda *et al.* reported that SRM after rosuvastatin treatment occurred approximately 1.9–2.7-fold higher than after atorvastatin treatment (Sakaeda et al., 2011). Similarly, Mueller *et al.* reported that the overall hazard ratio for rosuvastatin was 1.17 compared to atorvastatin (Mueller et al., 2021). In line with other results, our study showed that rosuvastatin had a higher incidence of SRM than atorvastatin. Why rosuvastatin has a higher incidence of SRM than atorvastatin is not clear, but it might be related to the different pharmacokinetic and pharmacodynamic properties of individual statins (Nikolic et al., 2020).

Co-administrated drugs may also affect musculoskeletal symptoms as drug-drug interactions increase the SRM risk. Interestingly, ezetimibe use with statins was correlated with SRM in the present study. Whether the concurrent use of ezetimibe with statins increases the SRM risk or not is controversial. Few studies have reported the effect of ezetimibe on the adverse events of statins, and the results did not have the same tendency. Cases of CK > 10 × ULN occurred in 0.1% of patients with combination statin and ezetimibe therapy, and in 0.4% of patients with statin monotherapy in clinical trials (Author Anonymous, 2023). In contrast, myalgia occurred more often in ezetimibe-statin groups than in statin-alone groups. The mechanism of ezetimibe-induced muscle symptoms is uncertain, but it might include myotoxicity caused by fatty acid oxidation impairment when ezetimibe and statins are administered together (Havranek et al., 2006). Unfortunately, we were unable to analyze the risk of the combination with niacin that could increase SRM risk because patients in this study did not take niacin (HPS2-THRIVE Collaborative Group, 2013). Fibrate could also increase the SRM risk (Graham et al., 2004), but there was no significant difference between the case and control groups in our study due to the small number of patients with fibrate (n = 5). However, our study showed that diuretic use increased SRM risk. According to Hopewell *et al.*, diuretic use with statins might be associated with a higher risk of SRM (Hopewell et al., 2020). This might be due to an electrolyte imbalance such as hypokalemia or physiological alterations including volume contraction (Mosenkis and Townsend, 2005).

Although we implemented the study on the relationship between SRM and CoQ10-related gene polymorphism, there were some limitations. First, this was the retrospective design based on past data. Second, it was conducted only on Koreans, which means further studies on Asians and other ethnic groups are needed. Third, the mechanisms of the SRM difference between rosuvastatin and atorvastatin as well as the pharmacological effect of ezetimibe on SRM, remain unclear. Fourth, CK levels of the case group were unaffected by gene polymorphisms, including rs4693075, rs11548336, and rs10849757. Considering that there were some missing values in CK levels, this needs to be validated through further studies. Fifth, we could not identify how different SNPs associated with lower or higher risk of statin-induced myopathy related to the molecular function of CoQ genes. Despite these limitations, this is the first study to show the influence of *COQ2*, *COQ3*, and *COQ5* genetic polymorphisms on SRM. As we showed the effects of clinical factors and genetic factors on SRM, this study might contribute to interpreting the cause of statin-induced musculoskeletal disorders and reducing the rate of statin withdrawal due to SRM.

## Data Availability

The data presented in the study are deposited in the Mendeley Data repository, https://data.mendeley.com/datasets/53454fx4hy/1.
