# Supplementary material for: Association between coenzyme Q 10-related genetic polymorphisms and statin-associated myotoxicity in Korean stroke patients
Source: Front Pharmacol. 2024 May 7;15:1358567. doi: 10.3389/fphar.2024.1358567 (PMC11106472; doi:10.3389/fphar.2024.1358567)
Supplement: Supplementary file 1 [file Table1.DOCX]

Supplementary Table 1. Effects of *COQ2, COQ3, COQ5,* and *COQ7* grouped genotypes on creatine kinase levels.

| **dbSNP rsID** | **Grouped genotype** | **Statin-related myotoxicity (n=56)** | ***p*** | **Control (n=632)** | ***p*** |
| --- | --- | --- | --- | --- | --- |
| ***COQ2*** |  |  |  |  |  |
| rs4693075 (G>C) | GG, CG | 152.3 ± 151.2 | 0.925 | 110.1 ± 60.3 | 0.444 |
|  | CC | 156.4 ± 118.9 |  | 105.6 ± 55.7 |  |
| rs121918233 (C>T) | CC | 154.3 ± 134.6 | NA | 106.9 ± 57.1 | NA |
| rs145182498 (G>A) | GG | 154.3 ± 134.6 | NA | 108.1 ± 57.5 | 0.006 |
|  | AA, AG | NA |  | 81.2 ± 36.4 |  |
| rs745504932 (C>T) | CC | 154.3 ± 134.6 | NA | 106.9 ± 57.1 | NA |
|  | TT, CT | NA |  | 90.0 ± 0.0 |  |
| rs121918230 (T>C) | TT | 159.4 ± 141.7 | 0.512 | 106.9 ± 55.9 | 0.837 |
|  | CC, CT | 92.0 ± 29.7 |  | 103.7 ± 44.3 |  |
| rs121918231 (C>T) | CC | 154.3 ± 134.6 | NA | 106.5 ± 56.7 | NA |
| rs121918232 (T>C) | TT | 154.3 ± 134.6 | NA | 106.9 ± 57.2 | 0.445 |
|  | CC, CT | NA |  | 81.7 ± 42.4 |  |
| rs34110644 (G>A) | GG, AG | 141.0 ± 143.8 | 0.468 | 108.9 ± 61.0 | 0.390 |
|  | AA | 173.4 ± 122.1 |  | 104.6 ± 52.7 |  |
| rs6849677 (T>C) | TT, CT | NA | NA | 141.2 ± 73.5 | 0.054 |
|  | CC | 154.3 ± 134.6 |  | 106.2 ± 56.6 |  |
| rs761785906 (G>A) | GG | 154.3 ± 134.6 | NA | 107.0 ± 57.1 | NA |
| rs867410805 (C>T) | CC | 154.3 ± 134.6 | NA | 107.0 ± 57.1 | NA |
| ***COQ3*** |  |  |  |  |  |
| rs6925344 (T>C) | TT, CT | 157.0 ± 61.5 | 0.909 | 105.4 ± 61.9 | 0.707 |
|  | CC | 152.8 ± 163.2 |  | 107.6 ± 55.3 |  |
| rs11548336 (T>C) | TT | 138.0 ± 65.5 | 0.479 | 102.5 ± 58.1 | 0.256 |
|  | CC, CT | 165.7 ± 167.5 |  | 108.8 ± 56.6 |  |
| rs146934336 (G>A) | GG | 154.3 ± 134.6 | NA | 107.0 ± 57.1 | NA |
| rs376598849 (C>T) | CC | 154.3 ± 134.6 | NA | 106.9 ± 57.1 | NA |
| rs200092962 (C>T) | CC | 154.3 ± 134.6 | NA | 106.9 ± 57.1 | NA |
| rs6912105 (A>G) | AA, AG | 155.8 ± 125.6 | 0.904 | 110.7 ± 61.4 | 0.015 |
|  | GG | 149.4 ± 169.8 |  | 98.7 ± 45.3 |  |
| rs769495529 (C>T) | CC | 154.3 ± 134.6 | NA | 107.0 ± 57.1 | NA |
| rs9389319 (A>C) | AA | 170.9 ± 73.9 | 0.725 | 104.2 ± 58.9 | 0.785 |
|  | CC, AC | 150.7 ± 145.2 |  | 107.1 ± 57.0 |  |
| rs9483838 (G>A) | GG | 158.8 ± 138.4 | 0.879 | 106.7 ± 57.5 | 0.926 |
|  | AA, AG | 151.8 ± 135.3 |  | 107.2 ± 56.9 |  |
| ***COQ5*** |  |  |  |  |  |
| rs3742049 (C>T) | CC | 161.5 ± 142.2 | 0.442 | 106.5 ± 56.5 | 0.582 |
|  | TT, CT | 114.8 ± 77.8 |  | 110.5 ± 60.4 |  |
| rs10849757 (G>A) | GG | 152.6 ± 64.3 | 0.976 | 107.5 ± 60.2 | 0.895 |
|  | AA, AG | 154.6 ± 142.7 |  | 106.7 ± 56.1 |  |
| rs14017 (T>C) | TT | 154.3 ± 134.6 | NA | 106.7 ± 56.9 | NA |
| rs144115488 (C>A) | CC | 154.3 ± 134.6 | NA | 107.0 ± 57.3 | NA |
|  | AA, AC | NA |  | 89.0 ± 0.0 |  |
| rs4766965 (G>A) | GG | 158.7 ± 139.0 | 0.485 | 104.8 ± 55.1 | 0.017 |
|  | AA, AG | 101.3 ± 43.1 |  | 140.1 ± 75.7 |  |
| rs1671766 (C>A) | CC, AC | 154.3 ± 134.6 | NA | 106.5 ± 56.0 | 0.691 |
|  | AA | NA |  | 111.8 ± 71.9 |  |
| rs758118388 (A>T) | AA | 154.3 ± 134.6 | NA | 107.0 ± 57.1 | NA |
| rs776253786 (C>T) | CC | 154.3 ± 134.6 | NA | 106.9 ± 57.1 | NA |
| ***COQ6*** |  |  |  |  |  |
| rs8500 (G>A) | GG, AG | 154.3 ± 134.6 | NA | 107.0 ± 56.2 | 0.782 |
|  | AA | NA |  | 104.3 ± 68.8 |  |
| rs2074930 (A>T) | AA | 125.1 ± 46.2 | 0.189 | 111.5 ± 60.6 | 0.082 |
|  | TT, AT | 179.3 ± 176.6 |  | 102.6 ± 52.4 |  |
| ***COQ7*** |  |  |  |  |  |
| rs138730205 (G>C) | GG | 156.6 ± 135.7 | NA | 107.5 ± 57.5 | 0.200 |
|  | CC, CG | 68.0 ± 0.0 |  | 87.7 ± 36.0 |  |
| rs4782202 (A>G) | AA, AG | NA | NA | 125.8 ± 57.2 | 0.151 |
|  | GG | 154.3 ± 134.6 |  | 106.2 ± 57.0 |  |
| rs11074359 (C>T) | CC, CT | 195.8 ± 179.1 | 0.335 | 105.9 ± 52.3 | 0.783 |
|  | TT | 143.6 ± 122.1 |  | 107.3 ± 59.3 |  |
| rs74841864 (G>A) | GG | 154.3 ± 134.6 | NA | 106.9 ± 57.1 | NA |
|  | AA, AG | NA |  | 99.0 ± 0.0 |  |
| rs77337400 (C>T) | CC | 140.3 ± 102.1 | 0.421 | 108.0 ± 57.5 | 0.135 |
|  | TT, CT | 218.1 ± 234.8 |  | 93.6 ± 50.0 |  |
| rs72777502 (C>T) | CC | 154.3 ± 134.6 | NA | 107.0 ± 57.1 | NA |
| rs864321686 (T>A) | TT | 154.3 ± 134.6 | NA | 106.9 ± 57.1 | NA |

NA: not available

Supplementary Table 2. Haplotype association analyses on statin-related myotoxicity.

| **Haplotype** | **Case** | | **Control** | | **OR**  **(95 % CIs)** | $\boldsymbol{x}^{\boldsymbol{2}}$ | ***p*** |
| --- | --- | --- | --- | --- | --- | --- | --- |
|  | **Frequency** | **Ratio** | **Frequency** | **Ratio** |  |  |  |
| *COQ2* haplotype^a^ |  |  |  |  |  |  |  |
| CA | 0.669 | 74.9 : 37.1 | 0.698 | 882.0 : 382.0 | 0.87 (0.58-1.32) | 0.402 | 0.526 |
| CG | 0.090 | 10.1 : 101.9 | 0.165 | 209.1 : 1054.9 | 0.50 (0.26-0.97) | 4.376 | 0.037 |
| GG | 0.240 | 26.9 : 85.1 | 0.134 | 169.4 : 1094.6 | 2.04 (1.29-3.24) | 9.510 | 0.002 |
| *COQ3* haplotype^b^ |  |  |  |  |  |  |  |
| GCC | 0.366 | 41.0 : 71.0 | 0.452 | 570.0 : 690.0 | 0.70 (0.47-1.04) | 3.101 | 0.078 |
| ACT | 0.420 | 47.0 : 65.0 | 0.406 | 511.0 : 749.0 | 1.06 (0.72-1.57) | 0.084 | 0.771 |
| GTT | 0.214 | 24.0 : 88.0 | 0.139 | 175.0 : 1085.0 | 1.69 (1.05-2.73) | 4.717 | 0.030 |
| *COQ5* haplotype^c^ |  |  |  |  |  |  |  |
| CA | 0.607 | 68.0 : 44.0 | 0.486 | 614.3 : 649.7 | 1.63 (1.10-2.43) | 6.037 | 0.014 |
| AG | 0.179 | 20.0 : 92.0 | 0.265 | 335.0 : 929.0 | 0.60 (0.37-0.99) | 4.018 | 0.045 |
| CG | 0.214 | 24.0 : 88.0 | 0.249 | 314.7 : 949.3 | 0.82 (0.51-1.32) | 0.666 | 0.414 |

^a^*COQ2* haplotype carrying rs4693075 (G>C) and rs34110644 (G>A); ^b^*COQ3* haplotype carrying rs9483838 (G>A), rs6925344 (T>C), and rs11548336 (T>C); ^c^*COQ5* haplotype carrying rs1671766 (C>A) and rs10849757 (G>A).
