# Supplementary figures and images for: Association between coenzyme Q 10-related genetic polymorphisms and statin-associated myotoxicity in Korean stroke patients
Source: Front Pharmacol. 2024 May 7;15:1358567. doi: 10.3389/fphar.2024.1358567 (PMC11106472; doi:10.3389/fphar.2024.1358567)

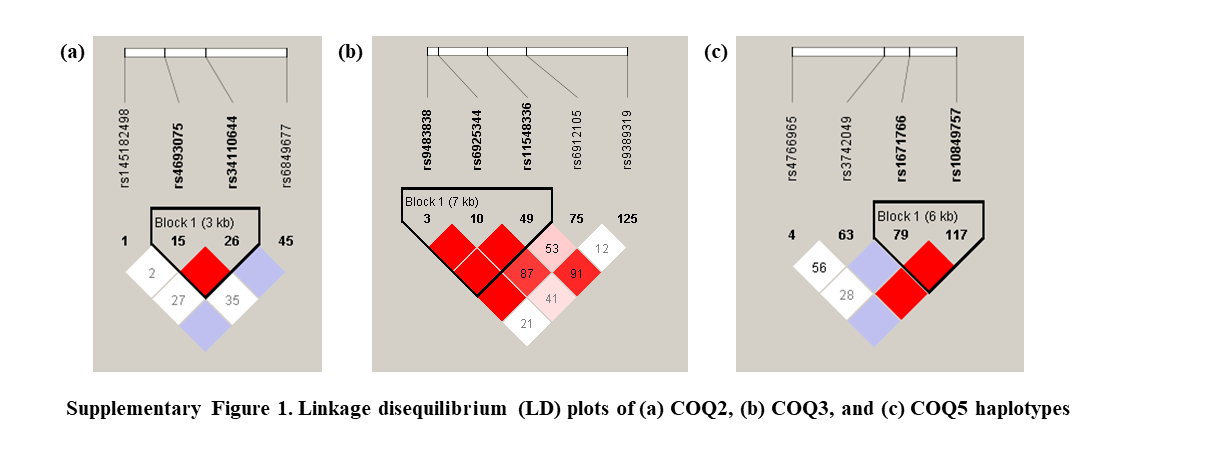

Supplement: Supplementary file 2 [file Image1.PNG]
